# Dynamic Decrease in Eosinophil After Intravenous Thrombolysis Predicts Poor Prognosis of Acute Ischemic Stroke: A Longitudinal Study

**DOI:** 10.3389/fimmu.2021.709289

**Published:** 2021-07-07

**Authors:** Dehao Yang, Honghao Huang, Yiyun Weng, Junli Ren, Chenguang Yang, Jianing Wang, Beibei Gao, Tian Zeng, Jingyu Hu, Wenjing Pan, Fangyue Sun, Xinbo Zhou, Guangyong Chen

**Affiliations:** ^1^ Department of Neurology, The Second Affiliated Hospital, Zhejiang University School of Medicine, Hangzhou, China; ^2^ Department of Neurology, The Third Affiliated Hospital of Wenzhou Medical University, Wenzhou, China; ^3^ School of the First Clinical Medical Sciences, Wenzhou Medical University, Wenzhou, China; ^4^ Department of Neurology, The First Affiliated Hospital of Wenzhou Medical University, Wenzhou, China; ^5^ Department of Internal Medicine, The Third Affiliated Hospital of Wenzhou Medical University, Wenzhou, China

**Keywords:** acute ischemic stroke, eosinophil, intravenous thrombolysis, inflammation, prognosis

## Abstract

**Background and Purpose:**

Blood eosinophil counts are thought to be associated with atherosclerosis in acute ischemic stroke (AIS) and AIS severity. We aimed to investigate 1): the temporal profile of eosinophil in AIS patients treated with recombinant tissue plasminogen activator (r-tPA); 2): The association between dynamic eosinophil and 3-month outcomes in different AIS etiologies; 3): incremental predictive ability of dynamic eosinophil adding to conventional model; and 4): the longitudinal change of neutrophil-to-lymphocyte ratio (NLR) and compared its prognostic value with eosinophils.

**Methods:**

A total of 623 AIS patients with intravenous thrombolysis in two hospitals were included. Blood samples were obtained on admission, within 24 h after an intravenous thrombolysis and on the seventh day. A multivariate logistic regression model with restricted cubic spline was performed to explore the association between dynamic eosinophil and a 3-month poor outcome. C-statistic, net reclassification improvement (NRI) and integrated discrimination improvement (IDI) were adopted to explore the incremental predictive ability.

**Results:**

Percent change in eosinophil counts after intravenous thrombolysis was median −25.00% (IQR −68.25%–+14.29%). Decrease in eosinophil >75% after intravenous thrombolysis was associated with 2.585 times risk for poor outcome and 13.836 times risk for death. However, the association were weak for patients outside of cardioembolic stroke. Adding eosinophil changes to a conventional model improved the discriminatory ability of poor outcome (NRI = 53.3%; IDI = 2.2%) and death (NRI = 101.0%; IDI = 6.9%).

**Conclusions:**

Dynamic decrease in eosinophil after intravenous thrombolysis predicts a 3-month poor outcome and death in AIS patients with r-tPA treatment and improved the predictive ability of conventional model. However, this result needs to be interpreted carefully in non-cardioembolic AIS patients.

## Introduction

Stroke, is a major cause of disability and mortality, in which acute ischemic stroke (AIS) accounts for nearly 80% (1). A fast and timely re-establishment of blood flow by intravenous thrombolysis using recombinant tissue plasminogen activator (r-tPA) is now recommended for AIS patients within 4.5 h of stroke onset (2). Ischemic stroke disrupts the balance that exits under quiescent conditions between coagulation and immune axes in the brain, causes not only local inflammation of ischemic lesions but also a peripheral immune response (3–6). At the same time, the infusion of r-tPA can cause changes in the immune environment *via* affecting the dynamic profile of peripheral leukocyte (7, 8). These immune alterations strongly influence the prognosis of AIS.

Though only about 1–8% of leukocytes in peripheral blood are eosinophils, they play an important role in the vascular innate immune system and contribute directly to the coagulation and fibrinolysis system (9). A few studies have previously looked at the role of eosinophils in AIS. Lower eosinophil count was observed in patients with myocardial infarction (10). Increased admission eosinophil was independently associated with the presence of aortic arch plaques in AIS (11). Interestingly, higher values of admission eosinophil were associated with mild stroke (12), and lower the risk of developing hemorrhagic transformation after intravenous thrombolysis (13). Besides, AIS patients with eosinopenia on the second day admission may have high infection rates, large volume of cerebral infarction and poor outcome (14). Meaningful conclusions from these studies are limited due to variable time points for blood sample collection, different treatment methods for AIS and heterogeneous populations. Therefore, the purpose of this study is to 1): identify the temporal profile of eosinophil in AIS patients treated with r-tPA; 2): evaluate the association between dynamic eosinophil and 3-month outcomes in different AIS etiologies; 3): find the incremental predictive ability of dynamic eosinophil adding to conventional model; and 4): evaluate the longitudinal change of neutrophil-to-lymphocyte ratio (NLR): a widely used biomarker representing an inflammatory status (15, 16), in our cohort and compared its predictive value with eosinophils.

## Materials and Methods

### Participants

**Figure 1:**
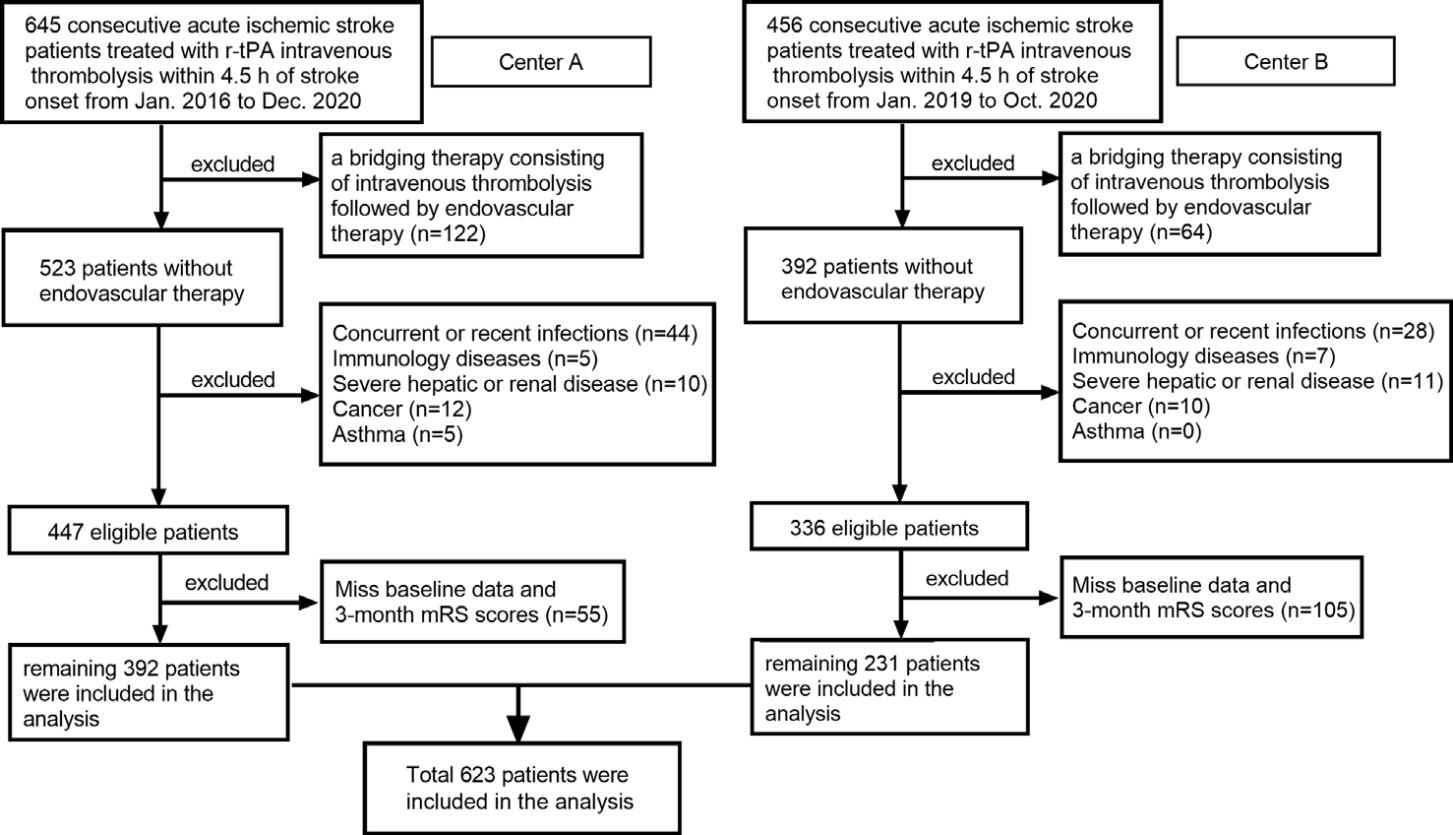
Flow chart for patients’ selection. Center A: Third Affiliated Hospital of Wenzhou Medical University; Center B: First Affiliated Hospital of Wenzhou Medical University.

In this retrospective cohort study, all consecutive patients with a diagnosis of AIS and treated with Intravenous r-tPA within 4.5 h stroke onset in the Third Affiliated Hospital of Wenzhou Medical University (Center A) from January 2016 to December 2020 and in the First Affiliated Hospital of Wenzhou Medical University (Center B) from January 2019 to October 2020 have been recruited to this study. Patients with 1): a bridging therapy consisting of intravenous thrombolysis followed by endovascular therapy; 2): concurrent or recent infections; 3): immunology diseases; 4): severe hepatic or renal disease; 5): cancer; 6): asthma; and 7): miss baseline data and 3-month mRS scores were excluded. A total of 623 patients remained for analysis (**Figure 1**).

### Data Collection

Patient’s demographic data (age, sex), risk factors (current smoking, history of hypertension, diabetes mellitus, atrial fibrillation, prior stroke), laboratory data (total cholesterol, triglycerides, high-density lipoprotein (HDL) cholesterol, low-density lipoprotein (LDL) cholesterol, glycated hemoglobin (HbA1c) values, leukocyte subtype counts), and clinical data (premorbid mRS, blood pressure, onset to needle time, National Institutes of Health Stroke Scale (NIHSS) score, stroke subtype) were obtained from the electronic medical records. Etiology of AIS patients were classified *via* the Trial of Org 10,172 in Acute Stroke Treatment (TOAST) criteria (17). Early ischemic changes on admission CT imaging were evaluated with the Alberta Stroke Programme Early CT Score (ASPECTS) (18). Blood samples were obtained on admission, within 24 h after intravenous thrombolysis and on the seventh day. In Center A, leukocyte subtype counts were analyzed using a Sysmex XT-1800i Automated Hematology Analyzer (Sysmex Corporation, Kobe, Japan). In Center B, leukocyte subtype counts were analyzed using a Sysmex XE-2100 Automated Hematology Analyzer (Sysmex Corporation, Kobe, Japan). Eosinophil changes after intravenous thrombolysis were calculated as (24 h-eosinophil counts − admission eosinophil counts)/admission eosinophil counts × 100%. The same formula was used to calculate the early changes of NLR. NIHSS scores were evaluated on admission, 24 h after intravenous thrombolysis and on the seventh day. Stroke outcome was evaluated using the modified Rankin Scale (mRS) which was collected on a phone interview at the 3-month follow-up. Good outcome was defined as the mRS score of 0–2 while poor outcome as the mRS score of 3–6.

### Statistical Analyses

Statistical analyses were performed *via* SPSS Statistics 25.0 software (SPSS Inc., Chicago, IL), MedCalc Statistical Software version 15.2.2 (MedCalc Software bvba, Ostend, Belgium; http://www.medcalc.org; 2015) and R version 4.0.3 (R Foundation for Statistical Computing, Vienna, Austria). Continuous variables were presented as the mean ± standard deviation (SD) or median (interquartile range, IQR) depending on the normality of distribution. Categorical variables are expressed as constituent ratios. The paired Wilcoxon signed-rank test was used to compare median eosinophil on admission and 24 h. The Spearman tests were used to analyze the correlation between admission eosinophil and the 24 h-eosinophil. Differences across multi-groups were assessed by means of the Chi-square test for categorical variables, and the one-way analysis of variance (ANOVA) or Kruskal–Wallis test for continuous variables. To explore the association between changes in eosinophil after intravenous thrombolysis and the 3-month outcome, univariable and multivariable logistic analyses were performed. Significant variables in the univariable analysis were entered in the multivariable model. Restricted cubic spline curves with four knots (at the fifth, 35th, 65th, and 95th percentiles) were further plotted to examine the linear relationship. C statistics, net reclassification index (NRI) and integrated discrimination improvement (IDI), were used to test the value of changes in eosinophil after intravenous thrombolysis to optimize the risk stratification for poor outcome and mortality. Statistical significance was set at two-tailed *p <*0.05.

## Results

### Characteristics of Enrolled Patients

A total of 623 eligible patients were recruited in our study, including 403 males (64.69%) and 220 females (35.31%). The mean age of the AIS patient was 67.36 years (SD ± 12.84). The median admission NIHSS score was 6 (IQR 4–10). According to TOAST classifications, 158 (30.02%) patients had cardioembolic stroke, 262 (42.05%) patients had atherothrombotic stroke, and 110 (17.66%) patients had lacunar infarction while 64 (10.27%) patients had AIS for other or undetermined reasons. The characteristics of the patients enrolled in the study are described in **Table 1**. In addition, characteristics of the patients before, excluded the missing data and the patients enrolled from different hospitals are described in **Supplementary Tables 1** and **2**.

**Table 1:** Characteristics of AIS patients According to Eosinophil Changes After Intravenous Thrombolysis.

Variable	Total (n = 623)	Increase or no change (n = 229)	0< decrease ≤25% (n = 88)	25%< decrease ≤50% (n = 102)	50%< decrease ≤75% (n = 66)	75%< decrease ≤100% (n = 138)	*p* value
Demographic data							
Age (years)	67.36 ± 12.84	66.28 ± 12.54	66.53 ± 12.44	66.17 ± 12.56	68.18 ± 12.44	70.16 ± 13.69	0.045
Sex (male, n.%)	403 (64.69)	157 (68.56)	63 (71.59)	68 (66.67)	36 (54.54)	79 (57.25)	0.043
Risk factors							
Current smoking (n.%)	166 (26.65)	65 (28.38)	33 (37.50)	30 (29.41)	11 (16.67)	27 (19.57)	0.011
Hypertension (n.%)	376 (60.35)	139 (60.70)	59 (67.05)	59 (57.84)	40 (60.61)	79 (57.25)	0.647
Diabetes (n.%)	113 (18.14)	43 (18.78)	25 (28.41)	19 (18.63)	12 (18.18)	14 (10.14)	0.015
Atrial fibrillation (n.%)	87 (13.96)	20 (8.73)	10 (11.37)	15 (14.71)	16 (24.24)	26 (18.84)	0.006
Previous stroke (n.%)	67 (10.75)	28 (12.23)	6 (6.82)	9 (8.82)	7 (10.61)	17 (12.32)	0.611
Laboratory findings							
Total cholesterol (mmol/L)	4.83 ± 1.13	4.81 ± 1.11	4.83 ± 1.26	5.09 ± 1.19	4.63 ± 1.00	4.73 ± 1.05	0.072
Triglycerides (mmol/L)	1.59 ± 1.22	1.74 ± 1.30	1.68 ± 0.90	1.57 ± 0.82	1.39 ± 0.94	1.37 ± 1.55	0.039
HDL cholesterol (mmol/L)	1.10 ± 0.26	1.06 ± 0.24	1.05 ± 0.27	1.14 ± 0.27	1.15 ± 0.27	1.15 ± 0.26	0.001
LDL cholesterol (mmol/L)	2.92 ± 0.90	2.89 ± 0.83	2.97 ± 1.01	3.13 ± 0.97	2.77 ± 0.90	2.85 ± 0.85	0.069
HbA1c values (%)	6.52 ± 1.41	6.46 ± 1.32	6.56 ± 1.36	6.70 ± 1.53	6.48 ± 1.66	6.45 ± 1.36	0.683
NLR (admission)	2.20 (1.48–3.44)	2.74 (1.74–4.33)	1.81 (1.36–2.66)	2.03 (1.40–3.04)	2.14 (1.28–3.17)	1.95 (1.37–2.34)	<0.001
NLR (24 h)	3.32 (2.30–5.00)	2.61 (1.95–3.50)	2.64 (2.05–3.63)	3.00 (2.41–3.92)	4.10 (3.10–5.80)	6.48 (4.83–9.72)	<0.001
NLR (percent changes, %)	42 (−5–+126)	0 (−36–+40)	37 (−11–+73)	40 (5–95)	94 (29–200)	231 (121–412)	<0.001
Blood pressure							
Systolic blood pressure (mmHg)	159.41 ± 24.78	157.50 ± 24.10	154.67 ± 21.01	162.20 ± 23.06	159.00 ± 30.16	163.86 ± 26.21	0.053
Diastolic blood pressure (mmHg)	89.55 ± 15.18	88.87 ± 14.39	87.99 ± 13.88	92.24 ± 17.46	87.43 ± 16.43	90.49 ± 14.77	0.198
Clinical characteristics							
Onset to treatment (min)	158 (122-204)	173 (130–211)	141 (104–188)	152 (120–209)	150 (113–197)	152 (128–199)	0.010
Premorbid mRS 0–1, n (%)	596 (95.67)	221 (96.51)	86 (97.73)	99 (97.06)	59 (89.39)	131 (94.93)	0.132
Admission NIHSS scores	6 (4–10)	5 (4–8)	5 (4–8)	6 (3–9)	7 (5–12)	10 (6–16)	<0.001
24 h-NIHSS scores	4 (2–8)	4 (2–5)	3 (2–7)	4 (2–8)	6 (4–11)	8 (4–14)	<0.001
7 d-NIHSS scores	3 (1–6)	2 (1–4)	2 (0–4)	3 (1–7)	4 (1–10)	5 (2–12)	<0.001
TOAST subtypes, n (%)							<0.001
Cardioembolism	187 (30.02)	48 (20.96)	22 (25.00)	25 (24.51)	26 (39.39)	66 (47.83)	
Large artery atherosclerosis	262 (42.05)	105 (45.85)	37 (30.68)	43 (42.15)	27 (40.91)	50 (36.23)	
Small artery occlusion	110 (17.66)	48 (20.96)	22 (25.00)	22 (21.57)	9 (13.64)	9 (6.52)	
Other/undetermined	64 (10.27)	28 (12.23)	7 (19.32)	12 (11.76)	4 (6.06)	13 (9.42)	

HbA1c, glycated hemoglobin; NLR, neutrophil-to-lymphocyte ratio; mRS, modified Rankin Scale; NIHSS, National Institute of Health Stroke Scale.

### Dynamic Eosinophil Distribution Within First 7 Days Symptom Onset

**Figure 2:**
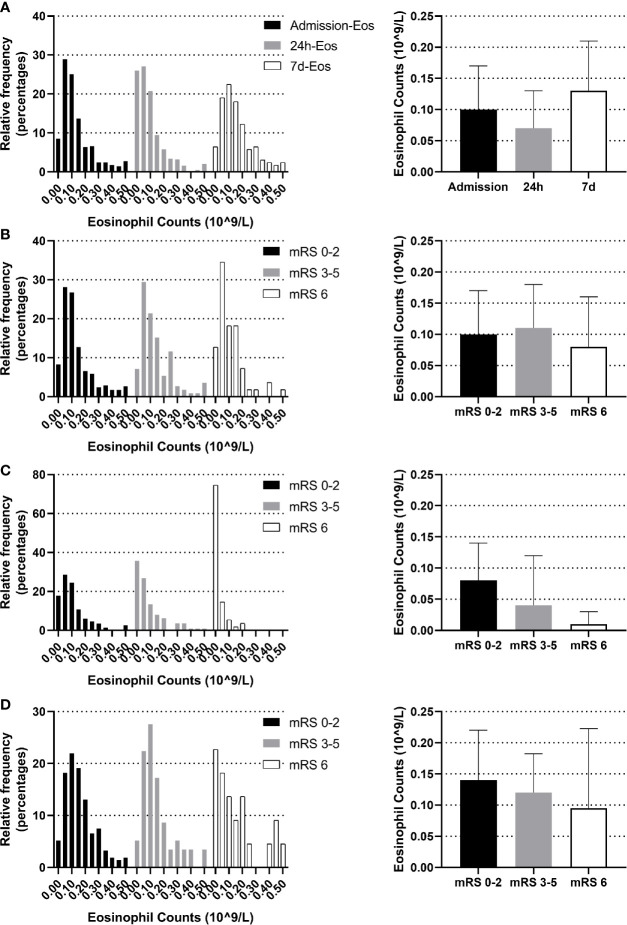
Temporal profile of eosinophil in AIS patients treated with r-tPA intravenous thrombolysis **(A)** Temporal profile of eosinophil on admission, within 24 h after intravenous thrombolysis and the seventh day **(B)** Distribution of admission eosinophil according to 3-month mRS scores. **(C)** Distribution of 24 h-eosinophil according to 3-month mRS scores. **(D)** Distribution of 7 d-eosinophil according to 3-month mRS scores. Frequency distribution charts (eosinophil >0.5 were included in the group with bin center = 0.5) and bar graphs (median and interquartile range) were displayed.

The blood eosinophil counts were collected on admission, 24 h and on the seventh day. The dynamic change of eosinophil was like a U shape. Comparing to admission eosinophil counts, patients had overall lower median eosinophil counts on 24 h (median 0.07 [IQR 0.02–0.13] vs. 0.10 [IQR 0.05–0.17] 10^9^/L, *p <*0.001). Eosinophil counts on the seventh day were available in 294 of the 623 patients (47.19%), with median 7 d-eosinophil counts of 0.13 (IQR 0.07–0.21) 10^9^/L (**Figure 2A**). The reasons for missing 7 d-eosinophil counts were either a moribund state or an early discharge. We didn’t identify the association between eosinophils and 3-month function outcomes using eosinophils collected on admission or the seventh day (*p* = 0.263, *p* = 0.290 respectively) (**Figures 2B, D** **)**. However, within 24 h after intravenous thrombolysis, patients with worse function outcomes tend to have lower eosinophil counts (median eosinophil 0.08 [IQR 0.04–0.14], 0.04 [IQR 0.01–0.12] and 0.01 [IQR 0–0.03] 10^9^/L in patients with the 3-month mRS 0–2, 3–5 and 6 groups respectively, *p <*0.001) (**Figure 2C**).

In addition, we found a strong and statistically significant positive correlation between admission-eosinophil counts and the 24 h-eosinophil counts (r = 0.558, *p <*0.001). To control for admission-eosinophil counts, we calculated the percent change in eosinophil counts after intravenous thrombolysis (median −25.00% [IQR −68.25–+14.29%]) and consequently patients were divided into five groups: increase or no change reference group (n = 229; increase n = 174; no change n = 55); 0< decrease ≤25% group (n = 88); 25%< decrease ≤50% group (n = 102); 50%< decrease ≤75% group (n = 66) and 75%< decrease ≤100% group (n = 138). Four (0.64%) patients with admission-eosinophil counts = 0, two patients with 24 h-eosinophil counts higher than 0 were assigned into increase or no change reference group while two patients with 24 h-eosinophil counts = 0 were assigned into 75%< decrease ≤100% group. Patients with greater decrease in eosinophils were significantly older, female, cardioembolic stroke, non-smokers, and had histories atrial fibrillation but not diabetes, with higher HDL cholesterol, lower triglycerides, lower admission NLR but higher 24 h NLR, shorter onset to treatment time, and higher admission, 24 h and the seventh day NIHSS scores (**Table 1**).

### Longitudinal Associations of Dynamic Eosinophil With Clinical Outcomes

**Figure 3:**
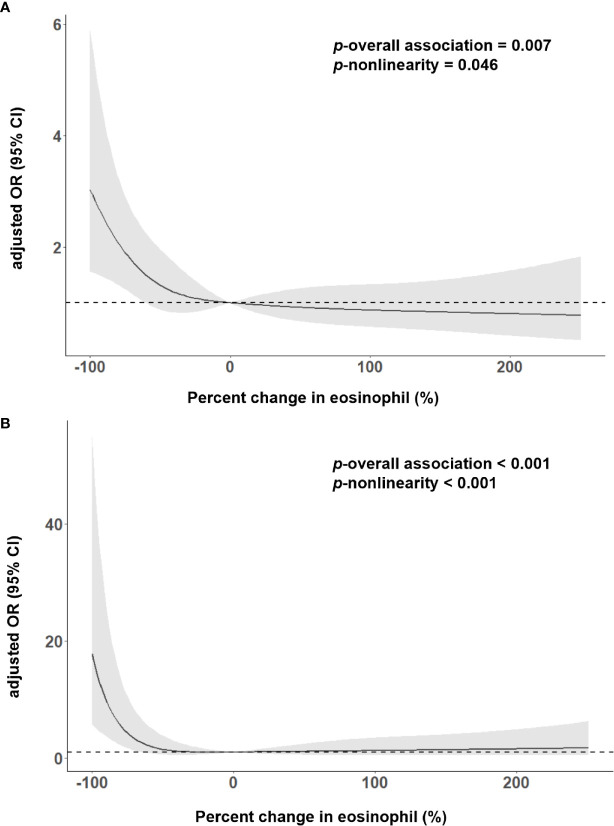
Adjusted association of eosinophil changes after intravenous thrombolysis with **(A)** 3-month poor outcome; **(B)** death, using multiple spline regression analyses with four knots (at the fifth, 35th, 65th, and 95th percentiles). The solid line indicates odds ratio, while the shadow indicates 95% CI. Reference point was 0. Data were adjusted for age, sex, current smoking, history of hypertension, atrial fibrillation, stroke, triglycerides and admission NIHSS score.

As shown in **Table 2**, 167 (26.81%) patients experienced the poor outcome (55 deaths and 112 major disabilities) during the 3-month follow-up. Patients with eosinophil counts 75%< decrease ≤100% had 6.441-fold increased risk for poor outcome and a 31.842-fold increased risk for death compared to increase or no change reference group. Adjustment for age, sex, current smoking, history of hypertension, atrial fibrillation, stroke and triglycerides slightly attenuated the association between Δeosinophil and AIS outcomes. In model 2, we observed an eosinophil count of 75%< decrease ≤100% after intravenous thrombolysis that led to 5.484 times risk for poor outcome and 28.069 times risk for death against the increase or no change reference group. This effect was consistent even after further adjusted for admission NIHSS scores in model 3, with OR = 2.585 (95% CI, 1.370–4.877) for poor outcome and OR = 13.836 (95% CI, 3.257–58.769) for death. In addition, in the continuous analyses, each 10% decrease in eosinophil after intravenous thrombolysis was associated with a 4.5% increase in the risk of poor outcome (OR = 1.045, 95%CI 1.007–1.084) and a 19.0% increase in the risk of death (OR = 1.190, 95%CI 1.071–1.322). Univariate logistic regression analyses of factors for adverse outcomes are displayed in **Supplementary Table 3**. A restricted cubic spline regression showed that a greater decrease in eosinophil after intravenous thrombolysis was associated with an increased risk of poor outcome and death and the depicted curves deviated significantly from linearity (*p* for non-linearity = 0.046 for poor outcome; *p* for non-linearity <0.001 for death) (**Figures 3A, B** **)**.

**Table 2:** Adjusted Odds Ratios of Adverse Outcomes at 3-month According to Eosinophil changes After Intravenous Thrombolysis.

Outcomes	Increase or no change (n = 229)	0< decrease ≤25% (n = 88)	25%< decrease ≤50% (n = 102)	50%< decrease ≤75% (n = 66)	75%< decrease ≤100% (n = 138)	Each 10% decrease in Eosinophils
Poor outcome (n.%)	34 (14.85)	13 (14.77)	24 (23.53)	23 (34.85)	73 (52.90)	
Crude Model	1	0.994 (0.497–1.987)	1.765 (0.983–3.167)	3.068 (1.644–5.724)	6.441 (3.929–10.560)	1.114 (1.075–1.155)
Model 1	1	0.987 (0.484–2.016)	1.841 (1.005–3.373)	2.890 (1.505–5.549)	5.971 (3.550–10.402)	1.105 (1.065–1.147)
Model 2	1	0.904 (0.429–1.904)	1.822 (0.970–3.423)	2.681 (1.350–5.326)	5.484 (3.150–9.547)	1.094 (1.053–1.137)
Model 3	1	0.802 (0.358–1.797)	1.659 (0.845–3.257)	1.880 (0.889–3.977)	2.585 (1.370–4.877)	1.045 (1.007–1.084)
Death (n.%)	3 (1.31)	4 (4.55)	1 (0.98)	6 (9.09)	41 (29.71)	
Crude Model	1	3.587 (0.786–16.365)	0.746 (0.077–7.258)	7.533 (1.830–31.006)	31.842 (9.628–105.313)	1.343 (1.225–1.472)
Model 1	1	3.664 (0.776–17.315)	0.782 (0.079–7.752)	6.772 (1.595–28.749)	28.069 (8.228–95.758)	1.316 (1.195–1.448)
Model 2	1	2.820 (0.522–15.224)	0.844 (0.084–8.511)	6.275 (1.422–27.690)	27.004 (7.590–96.075)	1.302 (1.176–1.443)
Model 3	1	2.781 (0.430–18.006)	0.855 (0.074–00.928)	5.455 (1.032–28.846)	13.836 (3.257–58.769)	1.190 (1.071–1.322)

Model 1, adjusted for age, sex.

Model 2, adjusted for age, sex, current smoking, history of hypertension, atrial fibrillation, stroke and triglycerides.

Model 3, adjusted for Model 2 and further adjusted for admission NIHSS scores.

### Results of Subgroup and Sensitivity Analysis

**Figure 4:**
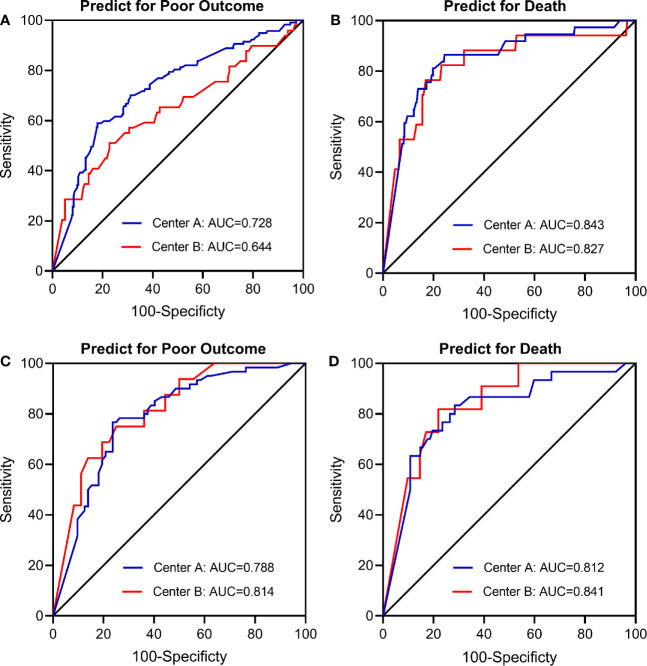
Receiver operating characteristic (ROC) curve for the value of eosinophil changes after intravenous thrombolysis to predict **(A)** Poor outcome in total patients (n = 623); **(B)** Death in total patients (n = 623); **(C)** Poor outcome in cardioembolic stroke only (n = 187); **(D)** Death in cardioembolic stroke only (n = 187). Center A: The Third Affiliated Hospital of Wenzhou Medical University; Center B: The First Affiliated Hospital of Wenzhou Medical University.

In the main analysis, we didn’t include ASPECTS into the multivariable logistic regression model due to the fact that ASPECTS were not available in Center B. In a first sensitivity analysis, we only selected AIS patients from Center A and additionally adjusted for the ASPECTS based on the conventional model. Results show that further adjustment for ASPECTS slightly attenuated the association between dynamic eosinophil and AIS outcomes, but the impact was relatively weak (**Supplementary Table 4**). Considering a total of 160 (20.43%) patients were excluded from the 783 eligible patients due to missing data, in the secondly sensitivity analyses, we chose the 690 patients with follow-up data and replaced the missing baseline and clinical data with the lowest 25th percentile, the 50th percentile, or the highest 25th percentile value of the 783 eligible patients. The association between dynamic eosinophil and AIS outcomes remained significant in the three models (**Supplementary Table 5**). The third sensitivity analysis excluded 19 patients with Tirofiban medications within 24 h after intravenous thrombolysis rendered very similar results as the main analysis (**Supplementary Table 6**). In addition, subgroup analysis was performed to explore the association between dynamic eosinophil and AIS outcomes in different TOAST classifications. Cardioembolic AIS patients with eosinophil counts 75%< decrease ≤100% had higher risk for poor outcome (OR = 11.453, 95%CI 2.661–49.283) and death (OR = 20.037, 95%CI 1.984–202.375) compared to increase or no change reference group. However, these associations were not observed in the AIS patients with TOAST classified large artery atherosclerosis, small artery occlusion and other/undetermined causes (**Table 3**). Finally, we compared the predictive values of eosinophils in Centers A and B using C-statistic. Results showed the continuous value of changes in eosinophil after intravenous thrombolysis had a prognostic significance for poor outcome and death in the both Centers A and B (**Figure 4** **)**. Compared to the area under the curve (AUC) for poor outcome in Center B, the AUC for poor outcome in Center A increased by 0.08, which may be due to fewer cardioembolic AIS in Center B. In addition, we restricted the analysis to patients with cardioembolic AIS and C-statistic was repeated. The AUC in the two centers were quite similar for poor outcome (Center A: 0.788, 95%CI [0.708–0.854]; Center B: 0.814, 95%CI [0.682–0.909]) and death (Center A: 0.812, 95%CI [0.735–0.875]; Center B: 0.841, 95%CI [0.714–0.928]).

**Table 3:** Adjusted odds ratios of adverse outcomes at 3-month according to eosinophil changes after Intravenous thrombolysis in different TOAST subtypes.

Outcomes	Increase or no change	0< decrease ≤25%	25%< decrease ≤50%	50%< decrease ≤75%	75%< decrease ≤100%	Each 10% decrease in Eosinophils
Poor outcome						
Cardioembolism	1	3.930 (0.668–23.123)	5.161 (0.956–27.590)	11.031 (2.129–57.153)	11.453 (2.661–49.283)	1.203 (1.087–1.333)
Large artery atherosclerosis	1	0.312 (0.086–1.134)	1.699 (0.700–4.120)	0.969 (0.313–3.001)	1.496 (0.599–3.737)	1.017 (0.977–1.059)
Small artery occlusion	1	0.603 (0.033–10.925)	NA	NA	NA	0.935 (0.767–1.141)
Other/undetermined	1	4.888 (0.337–71.010)	1.544 (0.100–23.885)	0.828 (0.003–225.626)	5.126 (0.528–46.242)	1.030 (0.954–1.112)
Death						
Cardioembolism	1	10.622 (0.650–173.592)	1.306 (0.049–34.813)	4.666 (0.359–60.680)	20.037 (1.984–202.375)	1.231 (1.054–1.437)
Large artery atherosclerosis	1	NA	NA	4.141 (0.403–42.588)	2.420 (0.283–20.678)	1.006 (0.900–1.125)
Small artery occlusion	NA	NA	NA	NA	NA	NA
Other/undetermined	NA	NA	NA	NA	NA	NA

Adjusted for age, sex, current smoking, history of hypertension, atrial fibrillation, stroke, triglycerides and admission NIHSS scores (Model 3 in **Table 2**).

NA due to lack of outcome events in this subgroup.

### Prognostic Value of Eosinophils and Neutrophil-to-Lymphocyte Ratio

**Figure 5:**
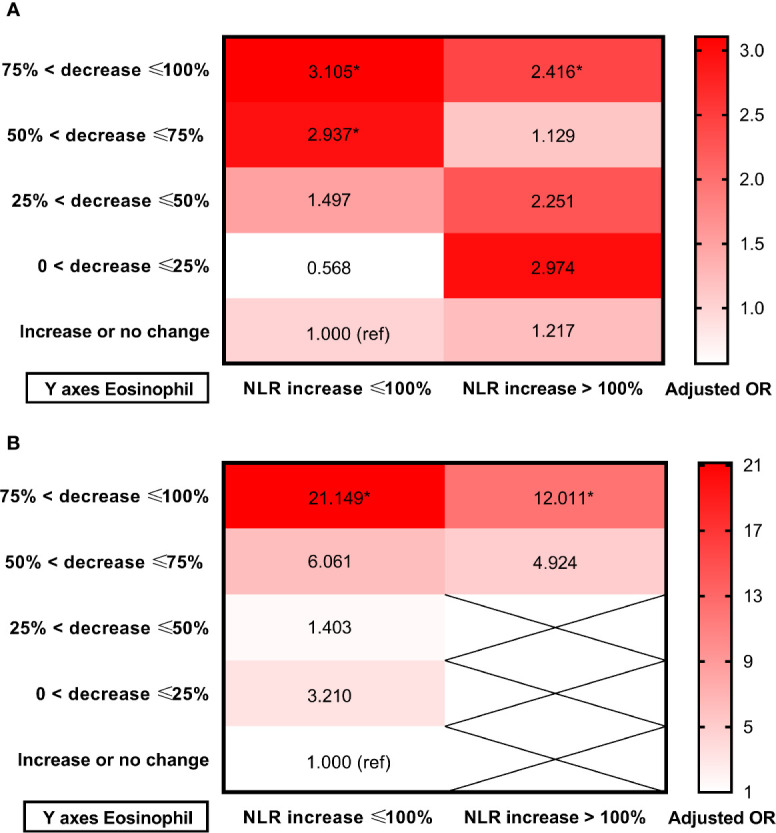
Heat maps of adjusted odds ratios for **(A)** Poor outcome; **(B)** Death in the 10 groups established using eosinophil and NLR changes after intravenous thrombolysis. Data were adjusted for age, sex, current smoking, history of hypertension, atrial fibrillation, stroke, triglycerides and admission NIHSS score (model 3). Asterisk indicates *p <*0.05. Total patients and outcome events in each group could be found in the **Supplementary Figure 2**.

Receiver operating characteristic (ROC) curves were employed to investigate the prognostic value of dynamic eosinophil and NLR changes after intravenous thrombolysis for adverse outcomes (**Supplementary Figure 1**). Areas under the curves (AUC) for poor outcome were 0.702 for dynamic eosinophil and 0.632 for dynamic NLR. The optimal cut-off values to separate patients experiencing poor outcome *vs.* good outcome were −57.14% for dynamic eosinophils with 57.23% sensitivity, 78.59% specificity and +97.27% for dynamic NLR with 49.10% sensitivity, 75.88% specificity. Besides, Areas under the curves (AUC) for death were 0.840 for dynamic eosinophil and 0.713 for dynamic NLR. The optimal cut-off values to distinguish mortality status were −61.54% for dynamic eosinophils with 85.19% sensitivity, 75.40% specificity and +124.35% for dynamic NLR with 61.82% sensitivity, 77.99% specificity. Thereby, for clinical practice, dynamic NLR were categorized into two groups: NLR increased ≤100% (n = 434), NLR increased >100% (n = 189). Multivariable logistic regression models showed that admission NLR, 24-h NLR and dynamic NLR were independent factors for poor outcome and death (**Supplementary Table 7**). Heat maps of adjusted ORs for poor outcome and death in the 10 groups established using eosinophil and NLR changes after intravenous thrombolysis showed that AIS patients with greater decrease in eosinophils were more likely to come to adverse outcomes regardless of the early change of NLR (**Figure 5**, **Supplementary Figure 2**).

### Incremental Predictive Ability of Dynamic Eosinophil

The new model shows an improvement in discrimination between good and poor outcomes after we added changes in eosinophil after intravenous thrombolysis into the conventional model (including age, sex, current smoking, history of hypertension, atrial fibrillation, stroke, triglycerides and admission NIHSS scores) with an AUC improved 0.06 (*p =* 0.304), NRI 53.3% (*p <*0.001) and IDI 2.2% (*p <*0.001). The discriminatory ability of the death was also improved in the new model (AUC improved 0.18; NRI 101.0%; IDI 6.9%) (**Table 4**). Similar results were found in cardioembolic stroke patients.

**Table 4:** Reclassification and discrimination statistics for adverse outcomes by eosinophils changes after intravenous thrombolysis in AIS patients.

	C-statistics		Continuous NRI		IDI	
	Estimate(95% CI)	*p* value	Estimate(95% CI)	*p* value	Estimate(95% CI)	*p* value
Predict for poor outcome (Total Patients, n = 623)						
Conventional model	0.847 (0.815–0.874)		Ref.		Ref.	
Conventional model + △Eosinophil	0.853 (0.822–0.880)	0.304	0.533 (0.358–0.709)	<0.001	0.022 (0.010–0.034)	<0.001
Predict for death (Total Patients, n = 623)						
Conventional model	0.932 (0.908–0.950)		Ref.		Ref.	
Conventional model + △Eosinophil	0.950 (0.929–0.966)	0.094	1.010 (0.772–1.248)	<0.001	0.069 (0.029–0.110)	<0.001
Predict for poor outcome (Cardioembolic Stroke Only, n = 187)						
Conventional model	0.886 (0.829–0.929)		Ref.		Ref.	
Conventional model + △Eosinophil	0.907 (0.854–0.946)	0.059	0.667 (0.389–0.944)	<0.001	0.053 (0.020–0.086)	0.001
Predict for death (Cardioembolic Stroke Only, n = 187)						
Conventional model	0.922 (0.872–0.957)		Ref.		Ref.	
Conventional model + △Eosinophil	0.942 (0.897–0.972)	0.067	0.971 (0.685–1.256)	<0.001	0.080 (0.033–0.126)	<0.001

The conventional model included age, sex, current smoking, history of hypertension, atrial fibrillation, stroke, triglycerides and admission NIHSS scores.

△Eosinophil enter the model in the form of categorical variable (5 groups displayed in **Tables 1–** **3**).

## Discussion

This study showed that: 1) Blood eosinophil count was a dynamic variable in AIS patients treated with r-tPA intravenous thrombolysis. Overall median eosinophil decreased significantly after intravenous thrombolysis; 2) Eosinophil within 24 h after intravenous thrombolysis but not on admission or 7 days was correlated with 3-month mRS scores; 3) Patients with greater decrease in eosinophils after intravenous thrombolysis were significantly cardioembolic stroke and had higher admission, 24 h and seventh day NIHSS scores; 4) In longitudinal analyze, dynamic decreased eosinophil after intravenous thrombolysis was associated with higher risk of poor 3-month outcome and death with a non-linear trend. However, the association were not statistically significant for patients outside of cardioembolic stroke; 5) In longitudinal analyze, dynamic increase in NLR after intravenous thrombolysis was associated with higher risk of poor 3-month outcome and death; 6) dynamic eosinophil had higher predictive ability than dynamic NLR for poor 3-month outcome and death; and 7) Adding eosinophil changes to a conventional model improved the discriminatory ability of poor outcome and death.

**Figure 6:**
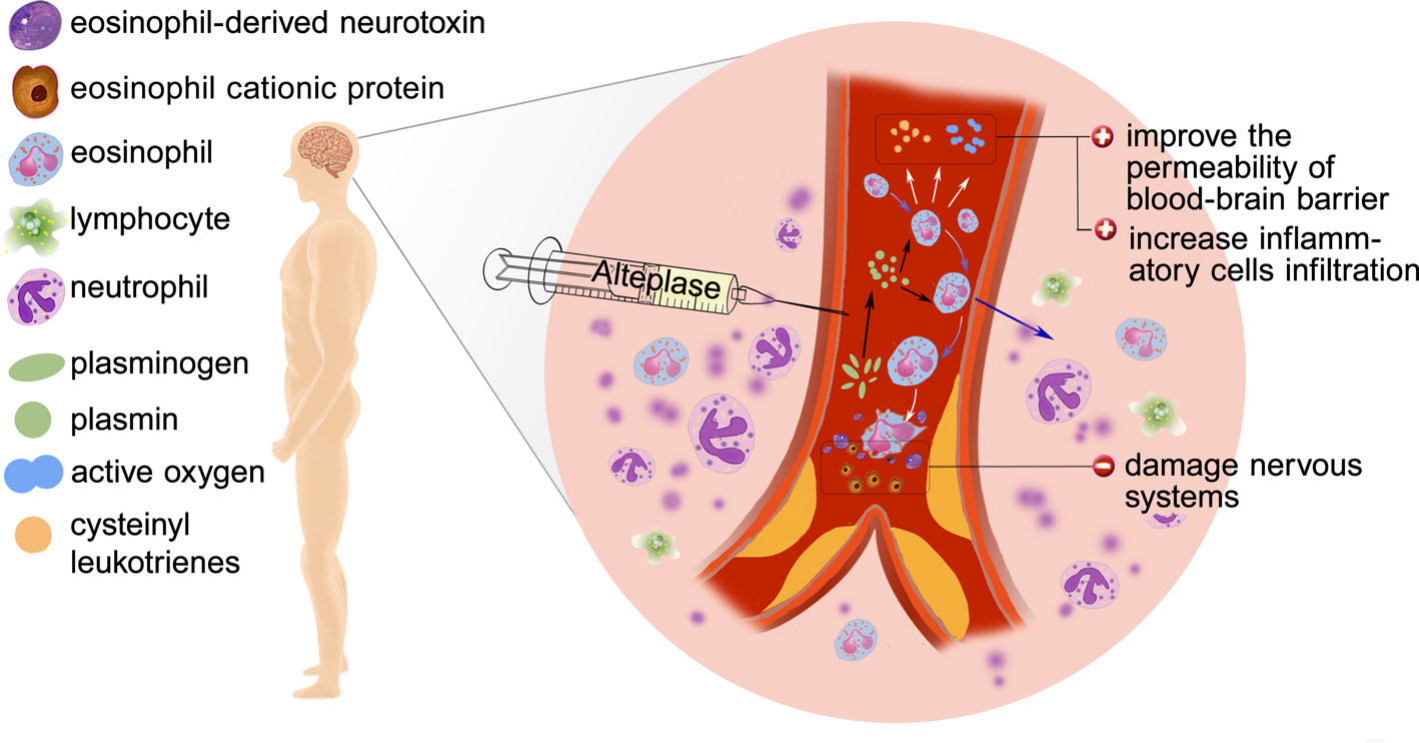
Possible mechanisms of poor outcome: Eosinophil infiltration, release of inflammatory factors, degranulation and release of toxic proteins.

The mechanism underlying the role of eosinophils in AIS outcome is still unclear and we hypothesize the following mechanism may explain the findings (**Figure 6**). Inflammation has been regarded as a key contributor to the AIS pathophysiology. Eosinopenia may be caused by acute inflammation through mechanism like sequestration of the eosinophil within an organ or eosinophils diffusing margination (19). Besides, eosinophils can synthesize and secrete more than 35 vital inflammatory and regulatory cytokines, growth factors, and chemokines, which participate in and regulate inflammation (20). It is worth noting that the infusion of r-tPA may contribute to the dynamic profile of eosinophils. *In vitro* studies found that the usage of r-tPA directly induced neutrophil degranulation (21), though whether r-tPA infusion will directly induce eosinophils degranulation is unknown, r-tPA administration can activate the fibrinolytic system and play a potentially supporting role in migration of eosinophils (22).

Whether eosinophil is beneficial or not depends on the particular condition. Eosinophils can produce nerve growth factor (NGF), vascular endothelial growth factor (VEGF), basic fibroblast growth factor (b-FGF) (23), which may play an important role in the functional recovery after AIS. Protease activated receptors (PARs) are known to be expressed by eosinophils and could be activated by proteases such as plasmin (9). Activation of PARs induced eosinophil shape change and lead to reactive oxygen species (ROS) and cysteinyl leukotrienes generation (24), which increases the permeability of blood–brain barrier (BBB) and contributes to leukocyte infiltration. Besides, human and mouse eosinophils degranulate upon stimulation and in inflammatory settings (25, 26). At the same time, activated eosinophils release cytotoxic proteins such as eosinophil cationic protein (ECP) and eosinophil-derived neurotoxin (EDN) which play a central role to the pathogenesis of AIS (27).

Previous study only collected the blood sample at a single time point (3, 12, 14, 28). Longitudinal association analysis is the advantage of our study. Besides, we provided a rather comprehensive view of the relationship between eosinophil and 3-month prognosis in AIS patients. Eosinophil on admission was not correlated with 3-month mRS scores of AIS patients, which may be due to the fact that the time from stroke onset to admission is limited to 4.5 h in patients with intravenous thrombolysis, and the change of eosinophils during this period is not significant. While on the seventh day, eosinophils of AIS patients were basically returned to the normal range. Our study demonstrated that association between changes in eosinophil after intravenous thrombolysis and 3-month prognosis were not statistically significant for patients outside of cardioembolic AIS. Patients with cholesterol crystal embolism (embolization of the contents of an atherosclerotic plaque from a proximal large-caliber artery), thicker aortic arch plaques with ulcer, or mobile component tend to have higher eosinophil with unknown mechanisms (11, 29), which may attenuate the association between eosinophils and AIS outcome in patients with atherothrombotic stroke. What’s more, our study suggested adding eosinophil changes after intravenous thrombolysis to conventional models could help clinicians to judge the prognosis of patients.

However, the present study have some existing limitations: 1) The data was retrospectively collected in this study and a total of 160 (20.43%) patients were excluded from the 783 eligible patients due to missing data. Besides, blood samples were only available at three time points and there were only 294 data for eosinophil on the seventh day due to moribund state or an early discharge; 2) Though patients were collected from two centers in this study, the results of the subgroup analysis was based on a small sample size, which may weaken statistical efficiency. In addition, ORs were unable to be obtained due to lack of events (poor outcomes or deaths) in some subgroups. Studies with larger sample size are warranted; 3) Though this study was observationally designed, residual confounders will exist and we had adjusted relatively strong confounding factors. Whether our results are universal, there is a need for more similar studies to prove such.

## Conclusion

A dynamic decrease in eosinophil after intravenous thrombolysis predicts a 3-month poor outcome and death in AIS patients with r-tPA treatment and adds prognostic information to the conventional model. However, this result needs to be interpreted carefully in non-cardioembolic AIS patients. We suggested the role of eosinophils in cardioembolic stroke and r-tPA intravenous thrombolysis merits attentions in future basic research.

## Data Availability Statement

The raw data supporting the conclusions of this article will be made available by the authors, without undue reservation.

## Ethics Statement 

The studies involving human participants were reviewed and approved by Ethics Committee of the Third Affiliated Hospital of Wenzhou Medical University and Ethics Committee of the First Affiliated Hospital of Wenzhou Medical University. Written informed consent for participation was not required for this study in accordance with the national legislation and the institutional requirements.

## Author Contributions

DY and GC conceptualized this work. DY and GC supervised the study. HH, YW, JR, CY, JW, BG, TZ, JH, WP, FS, and XZ: acquisition of data. DY, HH, YW, and JR performed the statistical analysis and interpreted data. DY, HH, and YW prepared the manuscript. DY, GC, HH, YW, JR, CY, JW, BG, TZ, JH, WP, FS, and XZ revised the manuscript. All authors contributed to the article and approved the submitted version.

## Conflict of Interest

The authors declare that the research was conducted in the absence of any commercial or financial relationships that could be construed as a potential conflict of interest.

## Abbreviations

AIS, acute ischemic stroke; r-tPA, recombinant tissue plasminogen activator**;** HDL, high-density lipoprotein; LDL, low-density lipoprotein**;** HbA1c, glycated hemoglobin**;** NIHSS, National Institutes of Health Stroke Scale**;** mRS, modified Rankin Scale**;** TOAST, Trial of Org 10,172 in Acute Stroke Treatment**;** NRI, net reclassification index**;** IDI, integrated discrimination improvement**;** AUC, area under the curve.
