# Supplementary material for: Dynamic Decrease in Eosinophil After Intravenous Thrombolysis Predicts Poor Prognosis of Acute Ischemic Stroke: A Longitudinal Study
Source: Front Immunol. 2021 Jul 7;12:709289. doi: 10.3389/fimmu.2021.709289 (PMC8293745; doi:10.3389/fimmu.2021.709289)
Supplement: Supplementary file 1 [file DataSheet_1.pdf]

Supplementary Table 1. Demographic and Clinical Characteristics of Patients before Excluded Missing Data.

	**Available cases**	**Values**
Demographic data		
Age (years)	783	67.15 ± 12.90
Sex (male, n.%)	783	502 (64.11)
Risk factors		
Smoking (n.%)	783	198 (25.29)
Hypertension (n.%)	783	460 (58.75)
Diabetes (n.%)	783	140 (17.88)
Atrial fibrillation (n.%)	783	103 (13.15)
Previous stroke (n.%)	783	87 (11.11)
Laboratory findings		
Total cholesterol (mmol/L)	718	4.76 ±.1.20
Triglycerides (mmol/L)	718	1.65 ± 1.31
HDL cholesterol (mmol/L)	718	1.10 ± 0.26
LDL cholesterol (mmol/L)	718	2.91 ± 0.89
HbA1c values (%)	658	6.51 ± 1.39
Eosinophil (admission)	733	0.10 (0.05-0.16)
Eosinophil (24 hours)	739	0.07 (0.02-0.13)
Eosinophil (percent changes, %)	695	-25.00 (-69.57-+14.29)
NLR (admission)	733	2.25 (1.50-3.63)
NLR (24 hours)	739	3.38 (2.33-5.33)
NLR (percent changes, %)	695	+41.03 (-5.43-+127.47)
Blood pressure		
Systolic blood pressure (mmHg)	747	160.16 ± 25.19
Diastolic blood pressure (mmHg)	747	89.95 ± 15.24
Clinical characteristics		
Premorbid mRS 0-1, n (%)	747	716 (95.85)
Admission NIHSS scores	752	6 (4-11)
TOAST subtypes, n (%)	748	
Cardioembolism		219 (29.28)
Large artery atherosclerosis		319 (42.65)
Small artery occlusion		129 (17.25)
Other/undetermined		81 (10.83)
Outcome measures	690	

Poor outcome, n (%)	193 (27.97)
Death, n (%)	69 (10.00)

Abbreviations: HbA1c, glycated hemoglobin; NIHSS, National Institute of Health Stroke Scale. NLR, neutrophil-to-lymphocyte ratio; mRS, modified Rankin Scale

Supplementary Table 2. Demographic and Clinical Characteristics of Included Patients from Different Centers.

	**Center A** (n = 392)	**Center B** (n = 231)	*p* value
Demographic data			
Age (years)	67.69 ± 13.16	66.80 ± 12.27	0.404
Sex (male, n.%)	259 (66.07)	144 (62.33)	0.346
Risk factors			
Smoking (n.%)	106 (27.04)	60 (25.97)	0.771
Hypertension (n.%)	245 (62.50)	131 (56.70)	0.154
Diabetes (n.%)	76 (19.39)	37 (16.02)	0.292
Atrial fibrillation (n.%)	76 (19.39)	11 (4.76)	< 0.001
Previous stroke (n.%)	47 (11.99)	20 (8.65)	0.195
Laboratory findings			
Total cholesterol (mmol/L)	4.63 ± 1.06	5.14 ±.1.17	< 0.001
Triglycerides (mmol/L)	1.53 ± 1.13	1.69 ± 1.34	0.101
HDL cholesterol (mmol/L)	1.09 ± 0.26	1.11 ± 0.25	0.265
LDL cholesterol (mmol/L)	2.94 ± 0.92	2.88 ± 0.86	0.445
HbA1c values (%)	6.51 ± 1.35	6.52 ± 1.53	0.484
Blood pressure			
Systolic blood pressure (mmHg)	160.00 ± 24.97	158.33 ± 24.45	0.426
Diastolic blood pressure (mmHg)	89.24 ± 15.27	90.11 ± 15.05	0.500
Clinical characteristics			
Onset to treatment (min)	153 (121-200)	167 (122-209)	0.062
Premorbid mRS 0-1, n (%)	374 (95.41)	222 (96.10)	0.680
ASPECTS	10 (9-10)	NA	
Admission NIHSS scores	7 (5-11)	5 (3-8)	< 0.001
TOAST subtypes, n (%)			< 0.001
Cardioembolism	133 (33.93)	54 (23.38)	
Large artery atherosclerosis	154 (39.29)	108 (46.75)	
Small artery occlusion	56 (14.29)	54 (23.38)	
Other/undetermined	49 (12.50)	15 (6.49)	

Abbreviations: HbA1c, glycated hemoglobin; ASPECTS: Alberta Stroke Program Early CT score; mRS, modified Rankin Scale; NIHSS, National Institute of Health Stroke Scale.

Center A: The Third Affiliated Hospital of Wenzhou Medical University;

Center B: The First Affiliated Hospital of Wenzhou Medical University

Supplementary Table 3. Univariable Logistic Regression of Parameters Associated with Functional Outcomes

	Poor Outcome		Death	
	Odds ratios (95% CI)	*p* value	Odds ratios (95% CI)	*p* value
Age (years)	1.062 (1.044-1.080)	< 0.001	1.117 (1.080-1.154)	< 0.001
Sex (male)	0.592 (0.412-0.851)	0.005	0.419 (0.240-0.720)	0.002
Current smoking	0.489 (0.313-0.766)	0.002	0.312 (0.131-0.743)	0.009
Hypertension	2.156 (1.462-3.180)	< 0.001	1.271 (0.711-2.271)	0.419
Diabetes	1.423 (0.916-2.211)	0.116	0.751 (0.344-1.636)	0.470
Atrial fibrillation	2.060 (1.286-3.301)	0.003	2.872 (1.525-5.407)	0.001
Previous stroke	2.154 (1.278-3.630)	0.004	1.720 (0.801-3.694)	0.164
Total cholesterol	0.968 (0.823-1.140)	0.698	0.874 (0.669-1.141)	0.321
Triglycerides	0.736 (0.687-0.923)	0.008	0.559 (0.351-0.890)	0.014
HDL cholesterol	1.464 (0.725-2.954)	0.288	1.656 (0.553-4.958)	0.367
LDL cholesterol	1.061 (0.866-1.302)	0.566	0.900 (0.635-1.255)	0.534
Systolic blood pressure	1.010 (1.003-1.018)	0.005	1.006 (0.995-1.017)	0.295
Diastolic blood pressure	0.995 (0.983-1.007)	0.390	0.998 (0.979-1.016)	0.805
ASPECTS	0.523 (0.411-0.665)	< 0.001	0.476 (0.360-0.629)	< 0.001
Admission NIHSS scores	1.250 (1.199-1.303)	< 0.001	1.287 (1.217-1.362)	< 0.001

Abbreviations: ASPECTS: Alberta Stroke Program Early CT score; NIHSS, National Institute of Health Stroke Scale.

ASPECTS was only available in Center A.

Supplementary Table 4. Adjusted Odds Ratios of Adverse Outcomes According to Eosinophil changes After Intravenous Thrombolysis (Center A with ASPECTS recorded)

Outcomes	Increase or no change (n = 135)	0 < decrease ≤ 25% (n = 64)	25% < decrease ≤ 50% (n = 61)	50% < decrease ≤ 75% (n = 42)	75% < decrease ≤ 100% (n = 90)	Each 10% decrease in Eosinophils
Poor outcome (n.%)	19 (14.07)	11 (17.18)	17 (27.87)	17 (40.48)	53 (58.89)	
Crude Model	1	1.267 (0.563-2.850)	2.359 (1.125-4.947)	4.152 (1.895-9.904)	8.745 (4.604-16.611)	1.152 (1.097-1.210)
Model 1	1	1.272 (0.552-2.931)	2.770 (1.252-5.825)	3.699 (1.633-8.379)	8.417 (4.291-16.509)	1.143 (1.087-1.202)
Model 2	1	1.243 (0.516-2.992)	2.763 (1.232-6.199)	3.589 (1.509-8.533)	7.854 (3.806-16.209)	1.130 (1.072-1.191)
Model 3	1	1.225 (0.475-3.157)	2.659 (1.123-6.295)	2.906 (1.147-7.358)	3.639 (1.588-8.335)	1.074 (1.020-1.132)
Model 4	1	1.304 (0.509-3.342)	2.590 (1.088-6.168)	2.686 (1.030-7.007)	3.541 (1.519-8.254)	1.072 (1.017-1.130)
Death (n.%)	2 (1.48)	3 (4.68)	0 (0)	4 (9.52)	28 (31.11)	
Crude Model	1	3.270 (0.533-20.078)	NA	7.000 (1.235-39.692)	30.032 (6.934-130.079)	1.402 (1.241-1.585)
Model 1	1	3.042 (0.530-21.069)	NA	5.590 (0.964-32.413)	25.777 (5.817-114.223)	1.377 (1.210-1.567)
Model 2	1	2.308 (0.298-17.856)	NA	4.947 (0.807-30.337)	21.505 (4.679-98.851)	1.356 (1.179-1.562)
Model 3	1	3.339 (0.278-40.029)	NA	9.199 (0.949-89.145)	11.967 (1.691-84.667)	1.235 (1.065-1.431)
Model 4	1	3.657 (0.318-42.100)	NA	5.047 (0.513-49.618)	8.059 (1.197-54.274)	1.177 (1.017-1.362)

Model 1, adjusted for age, sex.

Model 2, adjusted for age, sex, current smoking, history of hypertension, atrial fibrillation, stroke, triglycerides.

Model 3, adjusted for Model 2 and further adjusted for admission NIHSS scores

Model 4, adjusted for Model 3 and further adjusted for ASPECTS scores.

Supplementary Table 5. Adjusted Odds Ratios of Adverse Outcomes According to Eosinophil changes After Intravenous Thrombolysis in 690 Patients with Follow-up Data.

Outcomes	Increase or no change	0 < decrease ≤ 25%	25% < decrease ≤ 50%	50% < decrease ≤ 75%	75% < decrease ≤ 100%	Each 10% decrease in Eosinophils
Poor outcome						
Model 5	1	0.905 (0.423-1.932)	1.756 (0.423-1.932)	2.321 (0.927-3.325)	2.991 (1.686-5.309)	1.053 (1.019-1.087)
Model 6	1	0.883 (0.428-1.822)	1.449 (0.785-2.674)	1.717 (0.866-3.405)	2.754 (1.556-4.875)	1.038 (1.006-1.071)
Model 7	1	0.868 (0.436-1.727)	1.375 (0.735-2.571)	1.408 (0.691-2.867)	2.641 (1.497-4.661)	1.014 (0.990-1.038)
Death						
Model 5	1	5.496 (1.009-29.935)	2.117 (0.335-13.371)	10.201 (2.246-46.331)	15.261 (3.671-63.454)	1.193 (1.087-1.310)
Model 6	1	2.952 (0.826-10.553)	0.974 (0.226-4.118)	2.453 (0.691-8.704)	6.119 (2.203-16.996)	1.126 (1.044-1.213)
Model 7	1	1.689 (0.512-5.571)	0.542 (0.100-2.937)	1.592 (0.454-5.576)	4.863 (1.916-12.345)	1.055 (0.998-1.115)

Model 5, adjusted for Model 3; replaced missing data with the lowest 25th percentile of the entire cohort.

Model 6, adjusted for Model 3; replaced missing data with the 50th percentile of the entire cohort.

Model 7, adjusted for Model 3; replaced missing data with the highest 25th percentile of the entire cohort.

Supplementary Table 6. Adjusted Odds Ratios of Adverse Outcomes According to Eosinophil changes After Intravenous Thrombolysis (Excluded Patients with Tirofiban)

Outcomes	Increase or no change (n = 222)	0 < decrease ≤ 25% (n = 84)	25% < decrease ≤ 50% (n = 101)	50% < decrease ≤ 75% (n = 61)	75% < decrease ≤ 100% (n = 136)	Each 10% decrease in Eosinophils
Poor outcome (n.%)	33 (14.86)	11 (13.10)	23 (22.77)	21 (34.43)	71 (52.21)	
Crude Model	1	0.863 (0.414-1.798)	1.689 (0.932-3.059)	3.007 (1.578-5.729)	6.256 (3.795-10.314)	1.112 (1.073-1.154)
Model 1	1	0.858 (0.403-1.828)	1.784 (0.964-3.299)	2.726 (1.390-5.349)	5.966 (3.523-10.105)	1.104 (1.063-1.147)
Model 2	1	0.762 (0.345-1.685)	1.761 (0.929-3.339)	2.551 (1.257-5.178)	5.429 (3.097-9.516)	1.092 (1.051-1.135)
Model 3	1	0.655 (0.275-1.561)	1.587 (0.798-3.157)	1.758 (0.808-3.826)	2.536 (1.331-4.832)	1.042 (1.004-1.082)
Death (n.%)	3 (1.35)	4 (4.76)	1 (1.00)	5 (8.20)	41 (30.15)	
Crude Model	1	3.650 (0.799-16.667)	0.730 (0.075-7.105)	6.518 (1.512-28.098)	31.505 (9.520-104.259)	1.341 (1.222-1.471)
Model 1	1	3.774 (0.796-17.887)	0.779 (0.078-7.731)	5.704 (1.282-25.373)	29.657 (8.662-101.541)	1.321 (1.198-1.456)
Model 2	1	2.890 (0.535-15.614)	0.836 (0.083-9.436)	5.435 (1.181-25.001)	28.256 (7.927-100.722)	1.307 (1.178-1.449)
Model 3	1	2.894 (0.443-18.891)	0.824 (0.070-9.696)	4.590 (0.823-25.601)	14.910 (3.645-64.154)	1.194 (1.073-1.329)

Model 1, adjusted for age, sex.

Model 2, adjusted for age, sex, current smoking, history of hypertension, atrial fibrillation, stroke and triglycerides.

Model 3, adjusted for Model 2 and further adjusted for admission NIHSS scores.

Supplementary Table 7. Adjusted Odds Ratios of Adverse Outcomes at 3-month According to NLR

	Poor Outcome		Death	
	Odds ratios (95% CI)	*p* value	Odds ratios (95% CI)	*p* value
Admission NLR (per 1-point increase)				
Crude Model	1.060 (0.993-1.132)	0.079	1.100 (1.011-1.197)	0.027
Model 1	1.064 (0.993-1.141)	0.080	1.113 (1.014-1.222)	0.025
Model 2	1.070 (0.996-1.150)	0.064	1.123 (1.021-1.236)	0.017
Model 3	1.068 (0.988-1.154)	0.097	1.195 (1.068-1.338)	0.002
24 hours NLR (per 1-point increase)				
Crude Model	1.265 (1.190-1.345)	< 0.001	1.342 (1.250-1.440)	< 0.001
Model 1	1.250 (1.170-1.335)	< 0.001	1.312 (1.212-1.420)	< 0.001
Model 2	1.251 (1.168-1.339)	< 0.001	1.324 (1.217-1.441)	< 0.001
Model 3	1.172 (1.089-1.260)	< 0.001	1.278 (1.162-1.405)	< 0.001
Each 10% increase in NLR within 24h				
Crude Model	1.029 (1.018-1.140)	< 0.001	1.034 (1.021-1.047)	< 0.001
Model 1	1.026 (1.015-1.038)	< 0.001	1.028 (1.014-1.042)	< 0.001
Model 2	1.026 (1.015-1.038)	< 0.001	1.028 (1.014-1.043)	< 0.001
Model 3	1.015 (1.002-1.027)	0.020	1.017 (1.001-1.034)	0.040
NLR increase > 100% versus ≤ 100%				
Crude Model	2.927 (2.018-4.247)	< 0.001	5.139 (2.861-9.233)	< 0.001
Model 1	2.755 (1.858-4.087)	< 0.001	4.428 (2.364-8.293)	< 0.001
Model 2	2.819 (1.875-4.236)	< 0.001	4.336 (2.288-8.216)	< 0.001
Model 3	1.924 (1.208-3.064)	0.006	2.378 (1.142-4.952)	0.021

Model 1, adjusted for age, sex.

Model 2, adjusted for age, sex, current smoking, history of hypertension, atrial fibrillation, stroke and triglycerides.

Model 3, adjusted for Model 2 and further adjusted for admission NIHSS scores.
