# Supplementary material for: Dynamic Decrease in Eosinophil After Intravenous Thrombolysis Predicts Poor Prognosis of Acute Ischemic Stroke: A Longitudinal Study
Source: Front Immunol. 2021 Jul 7;12:709289. doi: 10.3389/fimmu.2021.709289 (PMC8293745; doi:10.3389/fimmu.2021.709289)
Supplement: Supplementary file 2 [file DataSheet_2.pdf]

Supplementary Figure 1

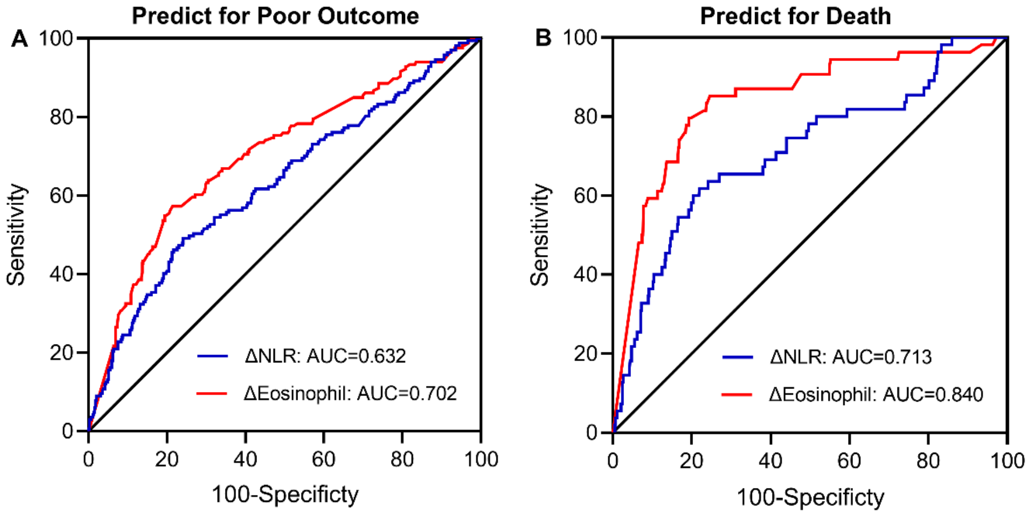

Receiver operating characteristic (ROC) curve for the value of eosinophil and NLR changes after intravenous thrombolysis to predict (A): Poor outcome; (B): Death. Dynamic eosinophil had higher predictive ability than dynamic NLR for poor 3-month outcome ($p = 0.002$) and death ($p < 0.001$).

Supplementary Figure 2

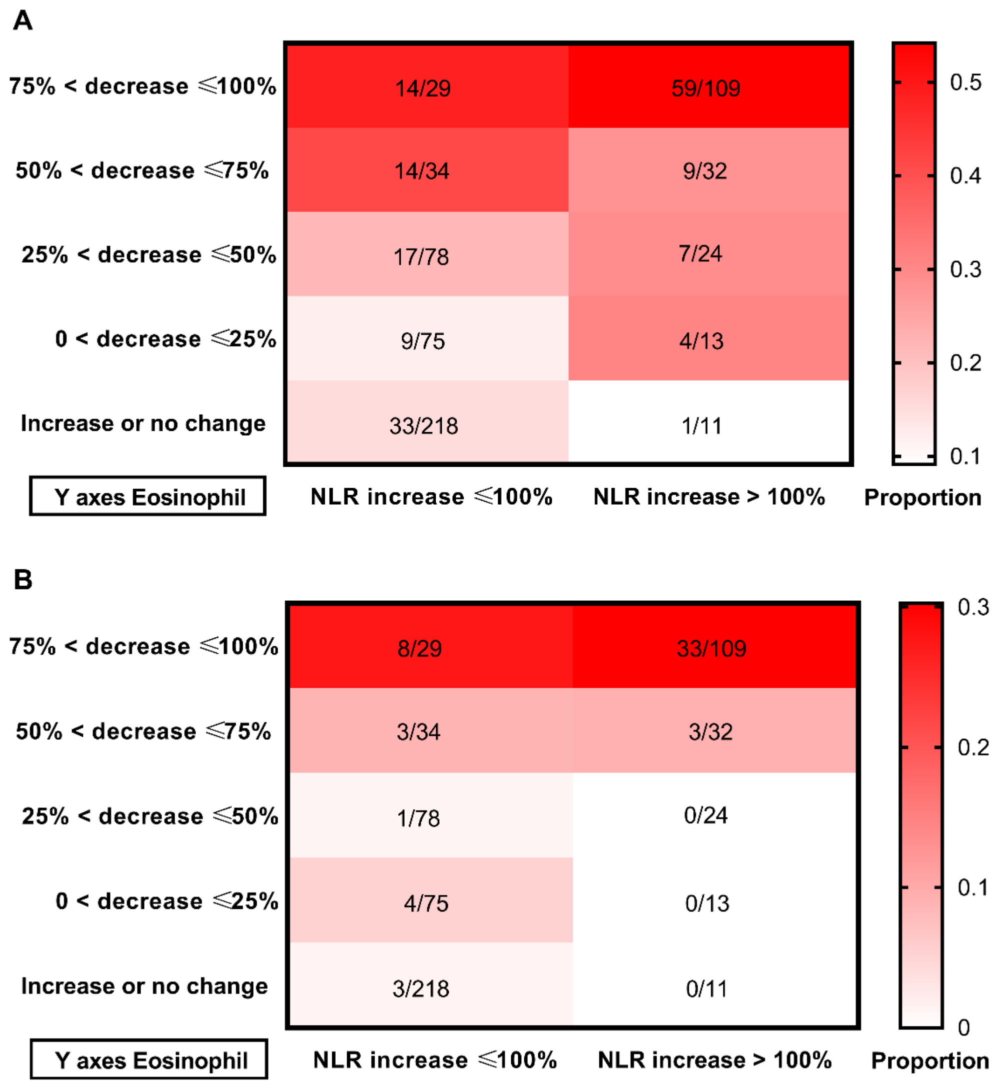

Heat maps of proportion for (A): Poor outcome; (B): Death in the 10 groups established using eosinophil and NLR changes after intravenous thrombolysis. Numbers in the right of / represent the total patients in the group while numbers in the left of / represent the outcome event cases in the group.
